# Evaluation of a Picture-Based Test for the Assessment of Gelotophobia

**DOI:** 10.3389/fpsyg.2017.02043

**Published:** 2017-11-21

**Authors:** Willibald Ruch, Tracey Platt, Richard Bruntsch, Róbert Ďurka

**Affiliations:** ^1^Department of Psychology, University of Zurich, Zürich, Switzerland; ^2^Department of Psychology, University of Wolverhampton, Wolverhampton, United Kingdom; ^3^Department of Psychology, Catholic University in Ružomberok, Ružomberok, Slovakia

**Keywords:** assessment, cartoons, gelotophobia, fear of being laughed at, perception of laughter, personality

## Abstract

This study examines whether coding open answers in a picture-based test, as to the extent they reflect the fear of being laughed at (i.e., gelotophobia), demonstrates sufficient validity to construct a semi-projective test for the assessment of gelotophobia. Previous findings indicate that cartoon stimuli depicting laughter situations (i.e., in the pilot version of the Picture-Geloph; Ruch et al., 2009) on average elicit fear-typical responses in gelotophobes stronger than in non-gelotophobes. The present study aims to (a) develop a standardized scoring procedure based on a coding scheme, and (b) examine the properties of the pilot version of the Picture-Geloph in order to select the most acceptable items for a standard form of the test. For Study 1, a sample of *N* = 126 adults, with scores evenly distributed across the gelotophobia spectrum, completed the pilot version of the Picture-Geloph by noting down what they assumed the protagonist in each of 20 cartoons would say or think. Furthermore, participants answered the GELOPH<15> (Ruch and Proyer, 2008), the established questionnaire for the subjective assessment of the fear of being laughed at. Agreement between two independent raters indicated that the developed coding scheme allows for objective and reliable scoring of the Picture-Geloph (mean of intraclass correlations = 0.66). Nine items met the criteria employed to identify the psychometrically most reliable and valid items. These items were unidimensional and internally consistent (Cronbach’s alpha = 0.78). The total score of this selection (i.e., the Picture-Geloph<9>) discriminated significantly between non-fearful, slightly, markedly, and extremely fearful individuals; furthermore, it correlated sufficiently high (*r* = 0.66; *r*_c_ = 0.79 when corrected for reliability of both measures) with the GELOPH<15>. Cronbach’s alpha (0.73) was largely comparable whereas the estimate of convergent validity was found to be lower in one (*r* = 0.50; *r*_c_ = 0.61; *N* = 103) of the two samples in Study 2. Combining all three samples (*N* = 313) yielded a linear relationship between the self-report and the Picture-Geloph. With the Picture-Geloph<9> and the developed coding scheme, an unobtrusive and valid alternative instrument for the assessment of gelotophobia is provided. Possible applications are discussed.

## Introduction

The concept of the fear of being laughed at (gelotophobia) was first developed in a clinical setting to describe and explain the negative perception of laughter by certain individuals (Titze, 2009). A core feature is that gelotophobes are deeply convinced that something essential is wrong with them and therefore it is inevitable that they make a funny impression on others (Ruch and Proyer, 2008). Based on this belief, they are—with a somewhat paranoid tendency—likely to feel that laughter is directed at them and thereupon misperceive it as ridicule (Platt, 2008; Titze, 2009).

When empirical research began, it became apparent that there are gradual interindividual differences in gelotophobia and a unidimensional approach (with the absence of the fear at the lowest end and extreme fear of being laughed at the upper end of a continuum) was established (cf. Ruch et al., 2014a). Since then, a number of studies have empirically validated the usefulness of the gelotophobia concept (Samson et al., 2011; Ivanova et al., 2012; Platt et al., 2013; Papousek et al., 2014; Durka and Ruch, 2015).

Fear and shame are the dominant negative emotions reported by gelotophobes (Platt and Ruch, 2009). When confronted with scenarios of either playful teasing or mean-spirited ridicule, extreme gelotophobes were found to respond with the same high amount of fear and shame in both scenarios. In contrast, non-gelotophobes showed a distinct, and less extreme, negative emotional response only to mean-spirited ridicule (Platt, 2008). There is also evidence that they blend the expressions of joy and contempt when decoding other’s facial displays of emotions (Hofmann et al., 2015; Ruch et al., 2015); i.e., for them a joyful face may hide an evil mind. On the level of physiological responses, it was found that cheerful and benign auditory laughter stimuli evoked a pronounced and more sustained decrease of the heart rate in gelotophobes (as compared to non-gelotophobes), which is regarded as indicating that gelotophobes perceive harmless laughter as a social rejection cue (Papousek et al., 2014). It was found that gelotophobes’ atypical responses to laughter could be triggered by different modalities of laughter, namely, by peculiarities in facial expression of smiles and laughter, the sound of laughter, and laughter-related body movement (Ruch et al., 2014b). As regards positive emotions, gelotophobes rated having a low inclination to joy (Platt and Ruch, 2009), and these lower levels of joy were also objectively measurable in facial expressions (Platt et al., 2013).

Taken together, gelotophobes are susceptible to “false alarms”; i.e., misinterpreting friendly and innocent laughter as malicious and threatening. They have a high propensity to fear and shame when confronted with laughter situations, and they have a low inclination to feeling and expressing joy. Therefore, as gelotophobia scores increase, individuals may detect real attempts of ridicule more readily, but this sensitivity comes at the price of being systematically misled by a biased perception when facing harmless or friendly social situations. When it comes to the assessment of gelotophobia, the outlined fear-typical tendencies can be targeted when aiming to measure the fear of being laughed at.

The fear of being laughed at was identified as a trait associated with a considerable range of psychological outcomes (e.g., social withdrawal, relationship status, experience of positive affect, life satisfaction, mental health; cf. Platt and Forabosco, 2012). In psychological humor research, gelotophobia was found to moderate the experience of humor situations: as gelotophobia scores increase, the valence of the response to smiling and laughter is inverted from a positive emotional response (e.g., amusement) to a negative response (e.g., fear and shame; cf. Ruch et al., 2014a). Consequently, the fear of being laughed at is worth considering when conducting experiments that involve the processing of humorous stimuli (cf. Fink et al., 2011) and should also be considered in clinical practice when dealing with patients suffering from social withdrawal due to disproportionate feeling of fear and shame in laughter situations (Platt et al., 2016).

### Assessing Gelotophobia

The standard self-report instrument is the GELOPH<15> (Ruch and Proyer, 2008), a 15-item self-report instrument utilizing a 4-point answer format. Cut-off points for slight, marked, and extreme fear of being laughed at were defined (Ruch and Proyer, 2008). While in non-clinical samples across the world typically the rate of slight fear is low (between 1.2 and 10%) and never exceeds 1% of the population (see overview in Platt and Forabosco, 2012), in clinical samples rates of 40% for slight fear and 10% for extreme fear were reported (Forabosco et al., 2009; Samson et al., 2011).

For the assessment of gelotophobia a multi-method approach was seen as desirable and therefore work on alternative methods of assessment, the structured interview (Platt et al., 2012) and the *Picture-Geloph*, a test with an open-ended answer format (Ruch et al., 2009) have been initiated. The advantage the structured interview is that it gives insight into the etiology of the problem and, in contrast to the questionnaire, does not impose on the participant the preconceived characteristics of gelotophobia. The Picture-Geloph uses 20 cartoons depicting fear-relevant situations, i.e., ambiguous social interactions showing people who were possibly being laughed at or could be seen as ridiculous. The test-taker is asked to fill in the empty thought or speech balloon and to write down what this person might be thinking or saying. The answers are then coded on a 5-point scale, ranging from -2 (i.e., answer reflects enjoyment of the situation) to +2 (i.e., answer reflects a fear of ridicule). Some of these cartoons include a laughing person (depicted by laugh utterances, or body movement) while others do not. Yet both are seen to be conducive to fearful answers. This is in line with the findings that gelotophobes also respond with increased fear and shame to harmless social situations (i.e., playful teasing; Platt, 2008) and that gelotophobes’ negative responses to laughter can be triggered by interpretation of visual and acoustic modalities (e.g., laugh sounds, facial expression, and body movement; Ruch et al., 2014b). Inasmuch as it (a) necessitates the attribution of one’s own experience of an ambiguous situation to another person, and (b) restricts the interpretation of the stimuli to the perspective of the protagonist and specifies the response by a thought or speech balloon (i.e., the task is not to associate freely), the Picture-Geloph may be classified as a semi-projective test^1^ (Greenstein and Tarrow, 1970; Gregory, 2004) comparable to the Rosenzweig Picture Frustration test (Rosenzweig, 1978). Compared to questionnaires semi-projective tests do have a lower face validity (and accordingly the measurement intention is not easily guessed) but unlike projective tests they can have good reliability (e.g., Sokolowski et al., 2000; Proyer, 2007).

A pilot study with the pilot version of the Picture-Geloph confirmed that gelotophobes are inclined to see mockery and laughing-at interactions in a variety of the social situations depicted by the cartoon stimuli whereas non-gelotophobes were inclined to respond with positive emotions instead (Ruch et al., 2009). While the results of the pilot study indicate that the rationale of the Picture-Geloph may be valid and promising, several steps are required before it could be used as a routine method for the assessment of the fear of being laughed at. The authors gave three recommendations to improve the test for further use: first, a larger pool of representative statements for the five steps of the rating scale needs to be developed to facilitate the coding process and to further enhance objectivity. Secondly, the importance of prior training of the coders is pointed out. In their study, the correlation between the total score of the Picture-Geloph and the Geloph<46> (i.e., the initial version of the GELOPH<15>; cf. Ruch and Proyer, 2008) was 0.72 for the trained coder but only 0.34 for the person less familiar with the concept. Thirdly, weaker items need to be identified and eliminated to eventually develop a reliable shorter standard form. In the pilot study a Cronbach’s alpha of 0.68 (for all 20 items) was reported, which increased to 0.74 after tentatively eliminating the eight items with corrected item total correlations of <0.25.

## Construction of the Picture-Geloph

The Picture-Geloph should be applicable for normal and clinical samples. Thus, for the construction of the instrument ideally a sample is needed that covers all levels of gelotophobia. Given that typically 90% in a sample are non-gelotophobes an oversampling of individuals from the higher end of the spectrum is needed, to have a sufficient size (of slight, marked, and extreme gelotophobes) to represent the entire spectrum and to allow for reliable group comparisons.

Using such a sample the construction project involves five steps. In the first step a coding scheme is developed and appraised to be further on used in the scoring of the test. A catalog of responses will be developed from a large pool of answers and they will be assigned a score (from -2 to +2) according. The coding scheme used by Ruch et al. (2009) will be taken as a basis but modified based on the responses of the present sample that will include more high-scorers. Also the theoretical rationale for the five stets of the answer scale will be improved. It will need to be verified that different trained coders converge in assigning the scores.

In the second step, the most fitting items will be identified and selected for the standard form of the Picture-Geloph. This is accomplished by engaging in two steps of analyses: (1) identifying the items where the coded answers match the GELOPH results both in terms of discriminatory power and the hedonic level of the answers, and (2) examining the psychometric properties of these items. Regarding the former, an item was considered ideal, if an item discriminates strongly among the five groups of people defined by no, slight, marked, and extreme fear of being laughed at (as verified by a significant linear trend in an ANOVA with *post hoc* tests yielding significant differences between adjacent groups), and where the no fear group (in the GELOPH) indeed on average yields affectively positive answers (e.g., < -0.5) and the average answers of the marked and extreme groups indicates a fearful answer (e.g., >1.0). Thus, an item is not considered optimal if it correlates highly with the GELOPH, but even marked gelotophobes interpret the situation as joyful, or if even the non-gelotophobes gives give answers to be coded as gelotophobic (e.g., when there is overt laughter and respondent acknowledges the fact that laughter is directed at him or her). As responses are rated to the degree to which they are fear-typical with absolute category labels (e.g., “*Explicitly fearing laughter*” or “*Neutral*”; see **Table 2**) it is reasoned that only such stimuli can be seen as conceptually valid which on average (a) elicit fear-typical responses (as identified by the coding scheme used) in gelotophobes but fear-atypical responses in non-gelotophobes, and (b) elicit more fear-typical responses as gelotophobia scores increase in different groups of gelotophobic participants. That is, even if there are *relative* differences between groups of individuals with different degrees of the fear, the item scores derived with the unified coding scheme are desired to reflect the *absolute* presence or absence of the fear. In the second step, the internal psychometric and structural properties of the items of the pilot version Picture-Geloph will be considered to refine the selection for the standard form by selecting items with the most acceptable loadings on the first unrotated principal component and corrected item-total correlations.

The third step is to determine the reliability and convergent validity of the newly developed standard version of the test (a) in terms of its correlation with the GELOPH<15>, and (b) in terms of whether it conceptually represent the variance of gelotophobia across *all defined levels* of the fear; i.e., whether the interpretation of the answers accurately represents the levels of gelotophobia as defined by the GELOPH (e.g., non-gelotophobes give non-fearful answers also at the level of the Picture-Geloph total score).

The fourth step will derive cut-off values for the score of the standard form of the test to enable the classification of subjects into non-fearful, slightly fearful, markedly fearful, and extremely fearful groups. The steps 1–4 will be undertaken in Study 1.

The fifth and final step in the construction is to find out whether the results from third and fourth step can be replicated in a further sample (Study 2).

## Study 1

Study 1 was designed to conduct the first four steps described above. Thus it pursues four aims, namely (a) to elaborate a coding scheme for the open answers required by the Picture-Geloph, (b) to select the most acceptable items from the pilot version of the Picture-Geloph in order to propose a standard form of the test, (c) to determine estimates of the reliability and convergent validity of the standard form, and (d) to suggest guidelines for its practical use in terms of cut-off values for the interpretation of the scores. In the sample, participants were included who provided a meaningful answer to each of the 20 items of the pilot version of the Picture-Geloph.

### Method

#### Participants

The sample was recruited worldwide over the Internet on a gelotophobia-dedicated website. It consisted of 126 adults, 50% male and 50% female; ages ranged from 18 years to 64 years (*M* = 28.5; *Md.* = 24; *SD* = 11.6). The sample consisted of 80.2% single, 6.3% cohabiting, 10.3% married, 1.6% divorced, and 1.6% widowed individuals.

Overall, an inspection of the averaged GELOPH<15> total scores confirmed that the recruitment strategy was successful. Participants’ gelotophobia scores ranged from 1.27 to 4.0 (*M* = 3.17, *SD* = 0.58). The cut-off points for gelotophobia (i.e., 2.5 for slight, 3.0 for marked, and 3.5 for extreme fear; Ruch and Proyer, 2008) were applied and yielded 11.9% (*n* = 15) individuals with no fear, and 17.46% (*n* = 22) with slight fear, 36.51% (*n* = 46) with marked fear, and 34.13% (*n* = 43) with extreme fear of being laughed at.

#### Instruments

*The GELOPH<15> (Ruch and Proyer, 2008)* is a questionnaire assessing the level of the fear of being laughed at (i.e., gelotophobia). It consists of 15 items in a 4-point answer format (1 = *strongly disagree* to 4 = *strongly agree*). A sample item is “When others laugh in my presence I get suspicious.” Cronbach’s alpha was 0.89 in the present sample, which is comparable to the English norm sample (α = 0.90; Platt et al., 2009). The GELOPH<15> has been adapted to a variety of languages across different cultural contexts (e.g., Proyer et al., 2009; Chen et al., 2011, 2013; Stefanenko et al., 2011).

*The Picture-Geloph (Ruch et al., 2009)*, in its pilot form, is a 20-item semi-projective test assessing the fear of being laughed at. Item scores are derived by coding the degree of the positive (i.e., joyful) vs. negative (i.e., laughing at) valence of participants’ written responses to cartoons. Cartoons are depicting social situations relevant to the fear of being laughed at with differing degrees of ambiguity. The 20 situations cover the following themes: (a) two persons might be laughing at or mocking a third one (five pictures), (b) a person is called to a situation in which he or she might make a fool of him- or herself (four pictures), (c) a person is in an unpleasant situation and/or might be laughed at or mocked by another person (seven pictures), (d) a person is criticized by another person (four pictures), and (e) a person is envious of others because they amuse themselves and he or she is not taking part (four pictures). The situations that are shown by the pictures are listed in **Table 1**.

**Table 1:** Descriptions of the cartoon pictures.

Item	Content	Type
1	A woman and a man are laughing (“Ha, Ha, Ha!”), a man with an empty thought balloon is watching.	a/e
2	A man on the phone is saying: “Can you speak more clearly? I cannot understand you” and a person in a phone booth with an empty speech balloon.	c/d
3	A man smiling at a woman with an empty speech balloon is saying: “Would you like to go out dancing with me? Heh-heh!”	b
4	A woman with an empty thought balloon is standing in front of a crowd of four sitting persons holding a paper in her hand.	c
5	A woman with a paper in her hand is pointing at a boy with an empty thought balloon saying “Heh! You can’t even solve such an easy task!”	d
6	A man is saying to another man with an empty speech balloon: “Will you join the Karaoke? It doesn’t matter if you can’t sing.”	b
7	Two boys are looking at each other laughing (“Hee hee!”), a girl with an empty thought balloon is looking away.	a/e
8	A man with an empty thought balloon is watching two persons on TV saying: “That’s really a very funny party!”	e
9	Two persons in an audience are laughing (“Hee Hee!”), a woman with a hat is sitting in the row in front of them.	a
10	A man is saying to another man with an empty speech balloon: “Relax and don’t always stand around in such an awkward manner.”	d
11	Four persons are sitting around a table, among them one with an empty thought balloon and one who is saying: “Let’s introduce ourselves. I will start.”	c
12	A man is smiling and looking at another man who obviously has tripped and fallen backward on the ground with an empty thought balloon.	c
13	Two women are standing close to each other, one of them saying “That is really interesting what you’re saying. I think that’s very amusing hee-hee!” A woman with an empty thought balloon is walking away.	a
14	A man is saying to another man with an empty speech balloon: “We want to perform a comedy onstage. Want to join?”	b
15	A woman is pointing at a girl sitting on a pupil’s desk with an empty thought balloon saying: “You did that really well.”	c
16	A boy is running away with a book from an old man with an empty thought balloon saying: “Heh, Heh!”	c
17	A man is saying to another man with an empty speech balloon: “Heh, Heh! Hello neighbor!”	c
18	A boy is saying to a man with an empty speech balloon wearing a hat, a beard and a necktie: “You really look funny!”	d
19	A woman is saying to a man with an empty speech balloon: “Come and join us for the party, it will be a good laugh!”	b
20	A man and a child are sitting at a table laughing (“HA! HA! HA!”), a man with an empty thought balloon and a glowing nose is watching.	a/e

*Type—type of situation: (a) two persons might be laughing at or mocking a third one (five pictures); (b) a person is called to a situation in which he or she might make a fool of him- or herself (four pictures); (c) a person is in an unpleasant situation and/or might be laughed at or mocked by another person (seven pictures); (d) a person is criticized by another person (four pictures), and (e) a person is envious of others because they amuse themselves and he or she is not taking part (four pictures).*

As **Table 1** shows, the cartoons depict either one person obviously interacting with a protagonist (designated by a thought or speech balloon), or ambiguous situations with one or more additional persons, in which the protagonist may—but may as well not—be concerned, or group situations that require the protagonist to do something (and one situation in which the protagonist is watching two persons interacting on TV).

#### Procedure

Data collection was administered via a website, specifically designed to collect the data and took place over the period of 6 years. Information websites such as Wikipedia, as well as media coverage of feature stories on gelotophobia were utilized to elicit participants by providing a URL that directed interested people to the website. In accordance with the University of Zurich’s code of ethics, assessment was conducted anonymously. Moreover, participants were able to quit at any time and were able to request to have any data removed from the database without any consequences or drawbacks. No personal identification information was taken but participants were offered a more in-depth assessment if they left a contact email address for where the participant’s gelotophobia score would be discussed in more general terms to help them gain insight into their own gelotophobia. After logging in on the website with a made up user name and a password, the participants first filled out the GELOPH<15>. After completing they filled out the pilot version of the Picture-Geloph. The study was conducted following the ethical guidance of the University of Zurich ethics checklist. Full disclosure and informed consent was provided prior to participation in the study by clicking on an “accept and continue”-link on the website. No participant had access to the study without agreeing. Altogether 403 participants visited the website and left data. For Study 1 only participants were used that had no missing data. As extreme gelotophobia is rare it was decided to retain the remaining 277 participants for potential use in Study 2.

A coding scheme for the appraisal of the valence of responses was developed (see **Table 2**). As **Table 2** shows, responses explicitly expressing the feeling or anticipation of being laughed at, mocked, made fun of, etc., were assigned to the most extreme scale value +2 (i.e., “explicitly fearing laughter”). Responses expressing negative emotion such as shame and fear, the impulse of withdrawal from the situation, the wish that the other person(s) would stop laughing, or feeling paranoid were rated as a “negative response, but not explicitly fearing laughter” (+1) to account for their more implicit indication of the fear of being laughed at. “Neutral” (0) values were assigned to responses that were not indicative of gelotophobic symptoms but, in turn, did not exhibit positive valence as well. A value of -1 (“slight enjoyment/engagement”) was assigned to responses that were not indicative of gelotophobic symptoms but expressing positive attributes, motives or emotions, and engagement in situations bearing the risk of being laughed at for the individual. Responses coded with a value of -2 (“full enjoyment/engagement”) met the criteria for -1 to a higher degree, that is, responses that reflected enthusiasm on top of positive attributes, motives or emotions, and indicators of behavioral engagement (vs. withdrawal)^2^.

**Table 2:** Scoring key for the coding of responses.

Score	General definition	Example responses for Item 1
+2	“*Explicitly fearing laughter*”: Responses that indicate fear of being laughed at by explicitly expressing the feeling or anticipation of being laughed at, ridiculed, made fun of, mocked, being seen as ridiculous, etc.	“Are they laughing at me?” “I need to get out of here they are laughing at me,” “They are probably mocking me,” “Why does everyone laugh at me?” “might be making fun of me.”
+1	“*Negative response, but not explicitly fearing laughter*”: Responses that are indicative of gelotophobic symptoms but do not explicitly express the feeling or anticipation of being laughed at (or ridiculed, made fun of, etc.) or might be as well indicative of a condition other than gelotophobia (such as social phobia), for example, expressing shame and fear, body symptoms related to fear and shame, the impulse of withdrawal from the situation, the wish that the other person(s) stop laughing, or feeling paranoid	“What are you laughing about?” “There something wrong with me (my clothes, hair,…),” “Am I that strange looking?” “I look ridiculous,” “Jerks,” “idiots,” “What’s so funny?” “Just ignore them,” “What did I do?” “Did I do something stupid?” “Confusion,” “paranoid,” “Look the other way and walk away. Walk, walk, walk,” “Please stop,” “shut up,” “They think I have a big nose,” “frigid numbness, insecurity,” “panic.”
0	“*Neutral*”: Neutral responses that are not indicative of gelotophobic symptoms but also have no positive valence in the sense of responses scored “-1” or “-2,” but also nonsense and humorous answers	“What are they talking about?” “Feel they talk about me,” “what is happening there?” “Hi, why are they wearing hats?” “Probably these people are sharing a funny story or joke,” “I’d like to join in on the laughter but I don’t know what to say.”
–1	“*Slight enjoyment/engagement*”: Responses that are not indicative of gelotophobic symptoms but expressing positive attributes, motives or emotions, but for some pictures also answers that reflect behavior that is contrary to a gelotophobic reaction (for example, joining a comedy performance group instead of avoiding it because of fear of being laughed at), or “healthy” reactions to a situation, less than a “-2”	“They look like their having fun,” “they are enjoying themselves. Good for them,” “I wish I knew that joke, seems funny.”
–2	“*Full enjoyment/engagement*”: Responses that match the definition of “+1” responses but in a higher degree (for example, joining a comedy performance group *enthusiastically*)	“They are having a good time as friends enjoying each others company,” “she likes him and he said something funny.”

*General definitions apply to all items. The assignment of example responses to score values varies between items. For example, an anger response will be assigned to a gelotophobic “+1” value if there is no obvious reason to take offense (for example, in item 1) but will be scored as a “-1” if the protagonist is insulted or explicitly bothered (for example, in item 10 or 18).*

A detailed definition of gelotophobia and a general characterization of gelotophobic persons, which was based on the state-of the art of the current findings of gelotophobia research, were utilized to train two coders. Furthermore, they were provided with the general definitions of rating scale steps shown in **Table 2**. Coders were blind to participants’ gelotophobia scores as obtained by the GELOPH<15>. One coder derived categories of responses item-wise and for every step of the rating scale and compiled them in a catalog. The analyses were based on the ratings of this coder. The other coder was used for estimating the level of convergence.

### Analysis and Results

The total scores derived by averaging each of the coders’ ratings over all 20 items of the pilot version of the Picture-Geloph were highly intercorrelated (*r* = 0.91) and both coders had a perfect blind agreement in 58% of responses rated. To attain an estimate of the objectivity and reliability of the coding procedure in terms of the degree of absolute agreement among measurements, intraclass correlations (ICCs) were computed between the two coders’ rating scores for the 20 items of the pilot version separately [by use of a two-way model (as the same two coders rated all responses), random effects (i.e., assuming that raters are replaceable), single measurements (i.e., analyzing individual item scores), and an agreement criterion (i.e., not adjusting the agreement for possible mean differences between the two coders to inform on the absolute objectivity of the rating procedure); cf. McGraw and Wong, 1996]. The results are given in **Table 3**. As **Table 3** shows, ICCs in the pilot version of the Picture-Geloph ranged from 0.39 to 0.83 with a median of 0.73 (and a mean of 0.67), showing that most of the variance in the ratings could be attributed to the participants (i.e., indicating an overall acceptable interrater agreement).

**Table 3:** Descriptive statistics, psychometric properties, interrater agreement, and the frequency distribution of the ratings of responses for the pilot version of the Picture-Geloph (Study 1).

Item	*M*	*SD*	–2 (%)	–1 (%)	0 (%)	+1 (%)	+2 (%)	ICC	CITC	FUPC
1	1.15	0.83	2.4	0.8	11.1	50.8	34.9	0.83	0.51	0.59
2	–0.07	0.94	0.0	46.8	14.3	38.1	0.8	0.56	0.42	0.50
3	0.49	0.99	5.6	11.1	19.8	55.6	7.9	0.59	0.43	0.51
4	0.46	0.81	0.8	8.7	42.9	38.9	8.7	0.59	0.53	0.61
5	0.43	0.67	0.0	7.1	46.0	43.7	3.2	0.70	0.29	0.36
6	0.34	1.16	12.7	7.9	21.4	48.4	9.5	0.78	0.42	0.51
7	0.87	0.87	1.6	2.4	27.8	43.7	24.6	0.76	0.45	0.53
8	–0.04	0.72	3.2	15.9	65.1	13.5	2.4	0.59	0.20	0.24
9	1.01	0.84	0.0	7.9	11.1	53.2	27.8	0.56	0.57	0.65
10	0.29	0.79	1.6	15.1	37.3	45.2	0.8	0.42	0.31	0.39
11	0.38	0.77	0.0	11.1	46.0	36.5	6.3	0.63	0.46	0.54
12	0.75	0.95	0.0	12.7	22.2	42.1	23.0	0.69	0.52	0.61
13	0.66	0.68	0.0	3.2	36.5	51.6	8.7	0.65	0.34	0.42
14	0.47	1.13	11.1	8.7	11.1	60.3	8.7	0.65	0.49	0.57
15	–0.07	1.03	11.9	18.3	37.3	30.2	2.4	0.83	0.37	0.44
16	0.77	0.87	0.8	5.6	30.2	42.9	20.6	0.73	0.41	0.48
17	0.03	1.03	10.3	14.3	42.9	27.0	5.6	0.74	0.37	0.44
18	–0.21	1.07	20.6	7.1	46.0	25.4	0.8	0.39	0.40	0.49
19	0.37	1.12	11.1	6.3	28.6	42.9	11.1	0.76	0.54	0.61
20	0.83	0.86	3.2	4.8	13.5	62.7	15.9	0.75	0.40	0.49
Md.	0.45	0.87	2.0	8.3	29.4	43.3	8.7	0.73	0.42	0.51

*N = 126. *M*, mean; *SD*, standard deviation; ICC, intraclass correlation between ratings of two different coders; CITC, corrected item-total correlation; FUPC, loading on the first unrotated principal component; *r*_rater_, intercorrelations between the raters; Items, ratings of responses to pictures 1–20; Md., median of columns. Frequencies of responses: “+2” = “explicitly fearing laughter,” “+1” = “negative response, but not explicitly fearing laughter,” “0” = “neutral,” “-1” = “slight enjoyment/engagement,” “-2” = “full enjoyment/engagement” (see **Table 2** for details).*

To ensure conceptual validity for the standard version of the Picture-Geloph, it was desired to arrive at a set of stimuli that gelotophobes respond to in a fear-typical way whereas non-gelotophobes respond without an indication of the fear of being laughed at or even in a positive way. As a second criterion, stimuli were defined as conceptually valid if responses in the groups of slight, marked, and extreme gelotophobes (as assessed with the GELOPH<15>) differed from each other in terms of different group means of scores within the Picture-Geloph^3^. Accordingly, we used participants’ gelotophobia scores as assessed with the GELOPH<15> to generate five groups with different degrees of gelotophobia in order to analyze which of the stimuli elicit responses that match the outlined criteria: (a) gelotophobes’ responses on average lie beyond a “neutral” threshold in terms of fear-typical responses whereas non-gelotophobes responses reflect absence of the fear in terms of positive responses, (b) group means of scores (i.e., codings of responses) show a linear increase along with the fear of being laughed at, and (c) among the group of gelotophobes, slight, marked, and extreme fear of being laughed at is reflected in higher scores among extreme gelotophobes than in the other two groups and higher scores among marked gelotophobes than in the group categorized as having a slight fear of being laughed at (according to the self-report measure).

Accordingly, Picture-Geloph single item score means were examined between the five groups with increasing gelotophobia scores separately (no fear, slight, marked, and extreme fear). Such items were selected (a) to which no-fear individuals on average responded to in a fear-atypical way (as indicated by negative group means), (b) plus to which marked fear individuals on average responded to in a fear-typical way (as indicated by positive group means), and (c) plus for which there was a constant increase in the item scores along with the gelotophobia level of the groups, i.e., for which there was no significant deviation from a linear trend (as tested using consecutive one-way ANOVAs with the GELOPH<15>, while employing gelotophobia level as the group factor and the Picture-Geloph items score as the dependent variable). These criteria led to a selection of nine items (i.e., items 1, 3, 4, 6, 7, 9, 11, 14, 19; see **Table 1** for content).

To inspect the internal psychometric properties of these items within the full pilot version of the scale, the corrected item-total correlations of the ratings of responses to the pictures were computed. Furthermore, a principal component factor analysis was performed on the intercorrelations of the ratings of responses to the 20 items in order to compute their loadings on their first unrotated principal component. There were six factors with eigenvalues exceeding unity (eigenvalues were 5.19, 1.50, 1.28, 1.22, 1.10, and 1.02). The first factor alone explained 25.95% of variance. To further inspect the properties of the single items, the means, standard deviations, and the frequencies of the different types of responses to every picture were computed. The results are given in **Table 3**.

**Table 3** shows that the most frequent coding was “negative response, but not explicitly fearing laughter” (+1), and more than 50% of the answers were yielded by this and the “explicitly fearing laughter” (+2) answer categories together. All of the selected nine items had acceptable loadings on the first unrotated principal component (>0.50) and acceptable corrected item-total correlations (>0.40) within the 20-item scale. These were taken to generate the standard form of the test, which will be labeled as the *Picture-Geloph<9>* in the remainder of this report.

#### Evaluation of the Standard form (Picture-Geloph<9>)

ICCs in the Picture-Geloph<9> ranged from 0.56 to 0.83 with a mean of 0.66, indicating that the overall interrater agreement was as acceptable as in the pilot version. A principal component factor analysis was performed on the intercorrelations of the ratings of responses to the nine items. There were two factors with eigenvalues exceeding unity (the first factor alone explained 37.56% of variance). The inspection of the scree plot (eigenvalues of the first two factors were 3.38 and 1.08) suggested that the items were unidimensional, which was substantiated by the results of a parallel analysis (Horn, 1965)^4^. Cronbach’s alpha for the nine-item scale was 0.78, indicating good internal consistency.

The mean total score of ratings of responses to the items of the Picture-Geloph<9> correlated moderately strong with the subjective self-report measure (GELOPH<15>) with *r* = 0.66, *p* < 0.001 (*r*_c_ = 0.79, when corrected for reliability of both measures as an estimate for the correlation of the true scores). The total score of the Picture-Geloph<9> was not correlated to participants’ age, *r* = -0.11, *p* = 0.243, but there was a trend for a correlation with gender, *r* = 0.17, *p* = 0.061 (with females tending to have higher scores than males).

To test whether individuals with higher degrees of self-reported gelotophobia would give more gelotophobic responses in the Picture-Geloph<9> than groups with lower degrees of subjective fear, Picture-Geloph<9> test scores were compared between four groups with different levels of gelotophobia (i.e., non-fearful individuals, individuals with slight, marked, and extreme fear of being laughed at). One-way ANOVAs with subsequent *post hoc* tests was conducted (Fisher’s least significant difference, LSD; effects with *p* < 0.05 are reported), with the Picture-Geloph<9> sum score as the dependent variable and the level of self-reported gelotophobia (as defined by the cut-off points of the GELOPH<15>) as a group factor. The results are given in **Table 4**.

**Table 4:** Frequency of types of responses and the sum score of the Picture-Geloph<9> as a function of level of gelotophobia (Study 1).

	Gelotophobia level (GELOPH<15>)							
	No		Slight		Marked		Extreme		Group comparisons		
*Picture-Geloph<9>*	*M*	*SD*	*M*	*SD*	*M*	*SD*	*M*	*SD*	*F*(3,122)	*p*	η^2^
*Frequency of responses*
“+2”	0.27_a_	0.59	0.50_a_	0.74	1.39	1.42	2.26	1.92	10.66	<0.001	0.21
“+1”	3.00_a_	1.81	3.91_a,b_	1.63	4.46_b,c_	1.72	4.79_b,c_	1.68	4.61	0.004	0.10
“0”	2.87_a_	0.92	2.82_a,b_	1.71	2.20_a,b,c_	1.34	1.65_c_	1.45	4.71	0.004	0.10
“-1”	1.53	1.19	0.95_a_	0.79	0.63_a_	0.80	0.21	0.47	12.75	<0.001	0.24
“-2”	1.33_a_	1.18	0.82_a_	1.05	0.33_b_	0.73	0.09_b_	0.48	11.12	<0.001	0.22
Sum score of ratings	–0.67	4.56	2.32	4.24	5.96	4.44	8.91	3.70	24.86	<0.001	0.38

*N = 126. *M*, mean; *SD*, standard deviation; η^*2*^, partial eta squared*. _a,b,c_*Means of one row sharing a subscript do not differ significantly from each other. Groups were defined by individuals’ GELOPH<15> scores: no gelotophobia <2.5, slight gelotophobia <3.0, marked gelotophobia <3.5, and extreme gelotophobia ≥3.5 (scale maximum = 4.0). “+2” = “explicitly fearing laughter,” “+1” = “negative response, but not explicitly fearing laughter,” “0” = “neutral,” “-1” = “slight enjoyment/engagement,” “-2” = “full enjoyment/engagement” (see **Table 2** for details)*.

As **Table 4** shows, Picture-Geloph<9> sum scores differed significantly as a function of the self-reported fear with a large effect size. *Post hoc* tests revealed that means of Picture-Geloph<9> scores differed among all groups, i.e., there were score differences in the full spectrum of self-reported gelotophobia. The effect sizes of *post hoc* comparisons were large and ranged between *d* = 0.68 [95% confidence interval (CI) = (0.009; 1.36); i.e., for the comparison between the fear-free group and the group with slight gelotophobia] and *d* = 2.43 [95% CI = (1.70; 3.17); i.e., for the comparison between the fear-free group and the group with extreme gelotophobia scores].

To gain a deeper insight into the kind of responses made in the Picture-Geloph<9> by the different gelotophobia groups, the number of answers in every step of the coding scheme was counted for every person. A one-way ANOVAs with subsequent *post hoc* tests (LSD; effects with *p* < 0.05 are reported) was computed with the frequency of responses as the dependent variable for each of the answer categories separately (e.g., “negative response, but not explicitly fearing laughter,” +1) and the gelotophobia level (as defined by the GELOPH<15> score) as a group factor. The results are also given in **Table 4**. As **Table 4** shows, the frequencies of the five answer categories differed significantly as a function of the GELOPH<15>-defined levels of gelotophobia (no, slight, marked, extreme), with medium to large effect sizes. On a descriptive level, as gelotophobia increased across the four groups, the prevalence of fear-atypical (i.e., “-1” and “-2”) answers and neutral responses decreased, whereas the prevalence of fear-typical (i.e., “+1” and “+2”) answers increased. However, although differences between the groups were in the expected direction, *post hoc* tests revealed that the means of frequencies of the different answer types did not differ significantly between all pairs of adjacent groups (cf. **Table 4**).

#### Deriving Cut-Offs for the Practical Use of the Picture-Geloph<9>

For the use of the newly designed standard version of the test, for example, for individual testing, the correspondence between the GELOPH<15> scores and the Picture-Geloph<9> scores were examined to derive cut-off points defining different levels of gelotophobia. The sum score of ratings (see **Table 4**; computed by adding the nine single item ratings) has a theoretical span of -18 to +18 and the actual values vary from a minimum of -9 to the maximum of 16 (*M* = 5.54, *SD* = 5.24). In the GELOPH agreeing to half of the items and disagreeing to the other half yields 2.5, and this is the cut-off score for where slight gelotophobia begins. A score of 0 has the same substance in the Picture-Geloph as this means there are as many gelotophobic interpretations as non-gelotophobic ones. A score lower than 0 indicates that at least most of the answers were neutral or fear-atypical. While 0–4 defines slight fear, 5–8 stands for marked fear, and 9 and more stands for extreme fear. This yields 19.8% with slight, 36.5% with marked, and 31% with an extreme expression of the rated fear, respectively (and 12.7% with no fear, i.e., <0). These group sizes largely corresponded to the groups defined by the established GELOPH<15> cut-off points (see section “Method”).

These scores also reflect differences in the GELOPH. A one-way ANOVAs with subsequent *post hoc* tests (LSD, effects with *p* < 0.05 are reported) with the GELOPH<15> mean as the dependent variable and the level of rated gelotophobia (as defined by the cut-off points of the sum score) as a group factor was conducted. Self-reported gelotophobia differed significantly as a function of the group factor as generated by the mentioned cut-off values [*F*(3,122) = 31.16, *p* < 0.001, η^2^ = 0.43). *Post hoc* tests revealed that means of self-reported gelotophobia differed among all groups as defined by the cut-off values (non-gelotophobes: *M* = 2.39, *SD* = 0.70, *n* = 16; slight fear group: *M* = 2.90, *SD* = 0.50, *n* = 25; marked fear group: *M* = 3.25, *SD* = 0.41, *n* = 46, and extreme fear of being laughed group: *M* = 3.57, *SD* = 0.26, *n* = 39). The effect sizes of *post hoc* comparisons were medium to large and ranged between *d* = 0.34 [95% CI = (-0.18; 0.85); i.e., for the comparison between the group with slight gelotophobia and the group with marked gelotophobia] and *d* = 1.92 [95% CI = (1.18; 2.65); i.e., for the comparison between the fear-free group and the group with extreme gelotophobia scores]. To account for the standard error of measurement, the CI was computed for the sum score of the Picture-Geloph<9> (accepting an alpha error at the 5% level) with a margin of error of 2.10. Consequently, as a heuristic (i.e., as slightly liberal) guideline, a CI of ±2 may be suggested when using the Picture-Geloph<9> for individual testing^5^. As the theoretical range of the scale is from -18 to +18 a CI of ±2 is acceptable.

## Study 2

As selection and validation of the Picture-Geloph<9> necessarily has been subject to the idiosyncrasy of the sample used in Study 1, an independent sample was used to cross-validate the findings. Accordingly, using an additional sample, Study 2 was designed to pursue the fifth aim of this paper: (a) to determine whether estimates of the reliability and convergent validity of the Picture-Geloph<9> are comparable to the ones found in Study 1, and (b) to find out whether the suggested guidelines for the practical use of the Picture-Geloph<9> (i.e., in terms of cut-off values for the interpretation of the scores) are useful also in this sample. Participants were included who provided a meaningful answer to each of the nine items of the newly developed standard form of the Picture-Geloph (i.e., the Picture-Geloph<9>).

### Method

#### Participants

Sample 2 consisted of 103 adults, 44.7% male and 55.3% female; ages ranged from 18 years to 60 years (*M* = 26.2; *SD* = 10.9). The sample consisted of 70.9% single, 12.6% cohabiting, 13.6% married, 2.9% divorced, and no widowed individuals.

Overall, an inspection of the averaged GELOPH<15> total scores confirmed that the recruitment strategy again was successful. Participants’ gelotophobia scores in Sample 2 ranged from 1.53 to 3.93 (*M* = 3.00, *SD* = 0.58). The cut-off points for gelotophobia (i.e., 2.5 for slight, 3.0 for marked, and 3.5 for extreme fear; Ruch and Proyer, 2008) were applied and yielded 24.3% (*n* = 25) individuals with no fear, and 75.7% (*n* = 78) gelotophobes. Among the latter there were 15.5% (*n* = 16) with slight fear, 39.8% (*n* = 41) with marked fear, and 20.4% (*n* = 21) with extreme fear of being laughed at. While the demographic characteristics of the sample were comparable to the sample used in Study 1, gelotophobia scores are lower in the present sample with more fear-free individuals and fewer extreme gelotophobes; i.e., variability was reduced.

Furthermore, a third sample (Sample 3) was used consisting of 84 adults (35% males; age: *M* = 23.7, *SD* = 0.9.7). Their GELOPH<15> scores were high on average (*M* = 2.95, *SD* = 0.65). There were 21.4% (*n* = 18) individuals each with no fear, slight fear, and extreme fear of being laughed at, respectively, while 35.7% (*n* = 30) had a marked fear of being laughed at.

#### Procedure

The procedure of Study 2 was identical to Study 1, except that this time the sample (Sample 2) was composed of individuals that had some missing data but answered all of the items of the newly developed 9-item standard form, i.e., the Picture-Geloph<9> (i.e., items 1, 3, 4, 6, 7, 9, 11, 14, 19, see **Table 1** for content). Cronbach’s alpha of the GELOPH<15> was 0.87 in Sample 2. Sample 3 answered to at least six of the nine items and Cronbach’s alpha of the GELOPH<15> was 0.89.

### Analysis and Results

ICCs were computed between the two coders’ rating scores for the nine items of the standard form separately in Sample 2 (again by use of a two-way model, assuming random effects, including single measurements, and an agreement criterion; cf. McGraw and Wong, 1996). ICCs in the Picture-Geloph<9> ranged from 0.48 to 0.75 with a mean of 0.61, indicating that the overall interrater agreement was somewhat lower than the one found in Study 1. To test for unidimensionality in Sample 2, a principal component factor analysis was performed on the intercorrelations of the ratings of responses to the nine items. The inspection of the scree plot (eigenvalues exceeding unity were 2.94, 1.29, and 1.10) suggested that the items were unidimensional, which was substantiated by the results of a parallel analysis (Horn, 1965)^6^. Cronbach’s alpha for the 9-item scale was 0.73, indicating that internal consistency was somewhat lower than in Study 1 (0.78).

The results for the individual items (descriptive statistics, frequency distribution of the ratings of responses, interrater agreement, factor loadings, corrected item total correlations, and correlations with the GELOPH<15>) for the final version of the test (i.e., *Picture-Geloph<9>*) in Sample 2 are computed and presented in **Table 5**.

**Table 5:** Descriptive statistics, the frequency distribution of the ratings of responses, interrater agreement, psychometric properties, and correlations with GELOPH<15> for the Picture-Geloph> (Sample 2).

Item	*M*	*SD*	–2 (%)	–1 (%)	0 (%)	+1 (%)	+2 (%)	ICC	CITC	FUPC	*r*_GELOPH_
1	1.03	0.79	1	2.9	14.6	55.3	26.2	0.72	0.47	0.61	0.41^∗∗^
3	0.54	0.87	2.9	9.7	24.3	56.3	6.8	0.49	0.20	0.30	0.23^∗^
4	0.27	0.81	1.9	9.7	54.4	27.2	6.8	0.62	0.33	0.49	0.19^∗^
6	0.38	1.16	12.6	8.7	15.5	54.4	8.7	0.64	0.57	0.73	0.31^∗∗^
7	0.76	0.91	1.9	2.9	35.9	35.9	23.3	0.75	0.40	0.58	0.28^∗∗^
9	0.69	0.90	0	12.6	22.3	48.5	16.5	0.58	0.43	0.60	0.21^∗^
11	0.17	0.85	4.9	10.7	49.5	32	2.9	0.48	0.40	0.53	0.28^∗∗^
14	0.42	1.18	14.6	5.8	9.7	63.1	6.8	0.58	0.52	0.68	0.32^∗∗^
19	0.19	1.09	9.7	16.5	23.3	45.6	4.9	0.62	0.36	0.51	0.29^∗∗^
Md.	0.42	0.90	2.9	9.7	23.3	48.5	6.8	0.62	0.40	0.58	0.28^∗∗^

*N = 103. *M*, mean; *SD*; standard deviation; ICC, intraclass correlation between ratings of two different coders; CITC, corrected item-total correlation; FUPC, loading on the first unrotated principal component; *r*_GELOPH_, correlations between item and the GELOPH<15>; Item, item number in Study 1; Md., median of columns. Frequencies of responses: “+2” = “explicitly fearing laughter,” “+1” = “negative response, but not explicitly fearing laughter,” “0” = “neutral,” “-1” = “slight enjoyment/engagement,” “-2” = “full enjoyment/engagement” (see **Table 2** for details). ^∗^*p* < 0.05 (two-tailed). ^∗∗^*p* < 0.01 (two-tailed).*

**Table 5** shows that again the most frequent coding was “negative response, but not explicitly fearing laughter” (+1), and more than 50% of the answers were yielded by this and the “explicitly fearing laughter” (+2) answer categories together. All of the selected nine items loaded positively on the first unrotated principal component (median of loadings >0.50) and positive corrected item-total correlations (median >0.40) within the 20-item scale. The items correlated significantly with the subjective assessment of the fear of being laughed at confirming that these items are suited to measure gelotophobia.

At the scale level, in Sample 2 the sum score of ratings of responses to the items of the Picture-Geloph<9> (*M* = 4.46, *SD* = 4.88) correlated moderately strong with the subjective self-report measure (GELOPH<15>) measure, *r* = 0.50, *p* < 0.001 (*r*_c_ = 0.61, when corrected for attenuation due to imperfect reliability of both measures). The total score of the Picture-Geloph<9> was correlated to participants’ age, *r* = -0.21, *p* = 0.034, and there were no gender differences, *p* = 0.275. In Sample 3, the Picture-Geloph<9> (*M* = 3.29, *SD* = 5.42) correlated highly with the GELOPH<15> measure, *r* = 0.65, *p* < 0.001, and there were no correlations with age (*r* = -0.08) or gender (*r* = 0.07). Thus, while the results will be better in the sample the items are selected than in replication samples, the high correlation in Sample 3 (similar to Sample 1) suggests that Sample 2 is the anomalous one (due to a lower variability of scores), and the results of Sample 1 can be trusted.

Again, one-way ANOVAs with subsequent *post hoc* tests was conducted (Fisher’s LSD; effects with *p* < 0.05 are reported) for combined Sample 2 and Sample 3, with the Picture-Geloph<9> sum score as the dependent variable and the level of self-reported gelotophobia (as defined by the cut-off points of the GELOPH<15>) as a group factor. The results are given in **Table 6**.

**Table 6:** Frequency of types of responses and the sum score of the Picture-Geloph<9> as a function of level of gelotophobia (Study 2; Sample 2 and Sample 3 combined).

	Gelotophobia level (GELOPH<15>)							
	No		Slight		Marked		Extreme		Group comparisons		
Picture-Geloph<9>	*M*	*SD*	*M*	*SD*	*M*	*SD*	*M*	*SD*	*F*(3,183)	*p*	η^2^
*Frequency of responses*
“+2”	0.16_a_	0.48	0.53_a_	0.78	1.31_b_	1.79	1.20_b_	1.17	8.70	<0.001	0.13
“+1”	2.59	1.46	4.34_a_	1.79	4.58_a_	1.81	4.91_a_	2.12	14.46	<0.001	0.19
“0”	3.42	1.64	2.65_a_	1.18	2.09_a_	1.38	2.33_a_	1.80	7.33	<0.001	0.11
“-1”	1.74	1.17	0.77_a_	0.96	0.66_a_	0.89	0.38_a_	0.70	17.28	<0.001	0.22
“-2”	1.09_a_	1.20	0.72_a_	1.02	0.36_b_	0.78	0.19_a_	0.48	8.70	<0.001	0.13
Sum score of ratings	–0.99	3.80	3.19	3.84	5.84_a_	4.92	6.54_a_	3.81	28.94	<0.001	0.32

*N = 187. *M*, mean; *SD*, standard deviation; η^*2*^, partial eta squared*. _a,b,c_*Means of one row sharing a subscript do not differ significantly from each other. Groups were defined by individuals’ GELOPH<15> scores: no gelotophobia <2.5, slight gelotophobia <3.0, marked gelotophobia <3.5, and extreme gelotophobia ≥3.5 (scale maximum = 4.0). “+2” = “explicitly fearing laughter,” “+1” = “negative response, but not explicitly fearing laughter,” “0” = “neutral,” “-1” = “slight enjoyment/engagement,” “-2” = “full enjoyment/engagement” (see **Table 2** for details).*

As **Table 6** shows, Picture-Geloph<9> sum scores differed significantly as a function of the self-reported fear with a large effect size. *Post hoc* tests revealed that means of Picture-Geloph<9> scores differed among all groups, except the last two (i.e., extreme and marked gelotophobia), which were in the right direction, however. Furthermore, the number of answers in every step of the coding scheme was counted for every person and subjected to one-way ANOVAs again. As **Table 6** shows, the frequencies of the five answer categories differed significantly as a function of the GELOPH<15>-defined levels of gelotophobia (no, slight, marked, extreme), with medium to large effect sizes. Again, as gelotophobia increased across the four groups, the prevalence of fear-atypical (i.e., “-1” and “-2”) answers and neutral responses decreased, whereas the prevalence of fear-typical (i.e., “+1” and “+2”) answers increased. Again, although differences between the groups were in the expected direction, *post hoc* tests revealed that the means of frequencies of the different answer types did not differ significantly between all pairs of adjacent groups (cf. **Table 6**).

As the three samples were recruited the same way a final analysis used all of them for a comparison. Studying all three subsamples together allowed for the most reliable inquiry of the form of the function linking the Picture-Geloph to the GELOPH<15>. As there were enough participants in terms of cell sizes, two groups of non-gelotophobes were distinguished, namely borderline and no fear. The 3 (samples) × 5 (level of fear of being laughed at) ANOVA yielded a main effect for level of gelotophobia, *F*(4,298) = 36.73, *p* < 0.001, η^2^ = 0.33, with no main effect of sample, *F*(2,298) = 1.89, *p* = 0.15, η^2^ = 0.01, and no sample × level of gelotophobia interaction, *F*(8,298) = 1.25, *p* = 0.27, η^2^ = 0.03. *Post hoc* tests revealed that all adjacent means were significantly different (*p* < 0.001), with non-gelotophobes (*n* = 19) scoring on the non-fearful side (*M* = -3.16; *SD* = 4.03) and borderline (*n* = 39) scoring in the indifference region (*M* = -0.19; *SD* = 3.50). Gelotophobes tend to give fearful answers, with the ones from slight (*n* = 56) gelotophobes being above the scale midpoint but reaching into the indifference region (*M* = 2.85; *SD* = 3.98), marked (*n* = 117) gelotophobes scoring clearly above the midpoint (*M* = 5.89; *SD* = 4.72), and extreme gelotophobes (*n* = 82) being highest with two standard deviations above the scale midpoint (*M* = 7.78; *SD* = 3.92). Except between the last two groups there is always an interval of three points between adjacent groups; i.e., there is a linear increase.

In the total sample with 313 adults the contingency between the coded levels of fear of being laughed at in the questionnaire and the picture test can be estimated. The cut-off values are applied and the cross-tabulation of scores (see **Table 7**) yielded a significant effect [χ^2^_(16)_ = 157.94, *p* ≤ 0.001] amounting to a correlation of 0.62.

**Table 7:** Crosstab of GELOPH<15> and Picture-Geloph<9> data, segmented into no fear, borderline, slight, marked, and extreme fear of being laughed at (Samples 1–3 combined).

Coded groups	No fear (1.0–2.0)	Borderline (2.1–2.5)	Slight (2.6–3.0)	Marked (3.1–3.5)	Extreme (3.6–4.0)	Total
No fear (≤-4.0)	8	6	5	6	0	25
	42%	15%	9%	5%	–	8%
Borderline (-3.9 to 0.0)	8	16	9	6	5	44
	42%	41%	16%	5%	6%	14%
Slight (0.1–4.0)	3	14	21	22	9	69
	16%	36%	38%	19%	11%	20%
Marked (4.1–8.0)	0	3	18	51	27	99
	–	8%	32%	44%	33%	32%
Extreme (>8.0)	0	0	3	32	41	76
	–	–	5%	27%	50%	24%
Total	19	39	56	117	82	313
	100%	100%	100%	100%	100%	100%

*N = 313.*

**Table 7** shows that the coded level of fear tends to correspond to each other. The scores in the diagonal are highest in both row and columns. The next highest frequencies can typically be found in the two adjacent cells. Interestingly, the gelotophobes with a marked fear of being laughed at have the largest variance in their scores, including having no fear at all in the Picture-Geloph. Thus, while there is no perfect overlap, the correspondence is striking and future studies need to see whether the Picture-Geloph has incremental validity over the GELOPH<15>.

### Discussion

For the present paper, two samples with a good distribution of slight, marked, and extreme gelotophobes were recruited, allowing for the construction, psychometric evaluation, and determining the validity of a standard form of the Picture-Geloph throughout the full spectrum of the fear of being laughed at. A coding scheme for the scoring of the test was developed and sufficient interrater agreement was found as an indicator of the objectivity and reliability of the standardized coding procedure. The compiled catalog of response-categories assigned to the respective scale values can be used as a reference in future studies as well as for supplementary information in individual testing in addition to the GELOPH (see **Table 2** for examples of reference answers to Item 1). Retest reliability estimates are still missing and hence it is not clear how much scores might fluctuate and depend on testing condition. However, internal consistency is high but not justifying sole administration. Hence further validation studies need to be conducted before routine use in individual testing is warranted.

In Study 1, the conceptually most valid items were selected and yielded acceptable psychometrical properties within the full scale (in terms of their loadings on the first unrotated principal component and their corrected item-total correlations). Thereby, it was ensured that the absolute meanings of the ratings of responses on average corresponded to individuals’ gelotophobia scores (i.e., as assessed with the established questionnaire, GELOPH<15>). Nine items were found which (a) elicit fear-typical responses (as identified by the coding scheme used) in gelotophobes but fear-atypical responses in non-gelotophobes, and (b) elicit more fear-typical responses as gelotophobia scores increase in different groups of gelotophobic participants. These items were used to generate a standard form of the test (i.e., the Picture-Geloph<9>). The items of the Picture-Geloph<9> were unidimensional and the reliability, as estimated by the internal consistency (Cronbach’s alpha = 0.78), was higher than compared to the initial study by Ruch et al. (2009), who reported a Cronbach’s alpha of 0.68 for the 20-item version and a coefficient of 0.74 for their 12-item proposal of a short form.

It was found that the Picture-Geloph was suitable to assess differences in the full spectrum of gelotophobia. As expected, individuals with higher degrees of self-reported gelotophobia gave more fear-typical responses than individuals with lower degrees of subjective fear, which was not only indicated by a substantial correlation between the scores of the Picture-Geloph<9> and the GELOPH<15>, but also by a comparison of Picture-Geloph<9> scores between groups with different levels of self-reported gelotophobia. Hence, the Picture-Geloph<9> can be regarded as suitable to validly assess gelotophobia in its full spectrum. Cut-off values for the sum score of the Picture-Geloph<9> (for the classification of subjects as non-fearful, slightly fearful, markedly fearful, or extremely fearful) were derived and found to separate the sample into four groups with differing GELOPH<15> score means.

In Study 2, the results of Study 1 were generally replicable, with some exceptions: (1) internal consistency and the correlation between the scores of the Picture-Geloph<9> and the GELOPH<15> (as an estimate of convergent validity) were numerically lower in Study 2 (for Sample 2 but not Sample 3), (2) the total score of the Picture-Geloph<9> was slightly correlated to participants’ age in Study 2 (only Sample 2), (3) the groups generated by the cut-off values for the sum score of the Picture-Geloph<9> that were derived in Study 1 did only in part differ from each other as to their mean level of self-reported gelotophobia in Study 2, indicating that the Picture-Geloph<9> was mainly suitable to discriminate between non-fearful and fearful individuals in the sample of Study 2. These deviations between the results of Study 1 and Study 2 may, partially, be attributed to the characteristics of one of the sample used in Study 2. Considering Sample 2 alone (a) the overall sample size was smaller, decreasing the power of statistical tests, (b) there was a smaller proportion of gelotophobes, and (c) especially extreme gelotophobes were less represented (as compared to Study 1), overall leading to a reduced variance in gelotophobia scores. The reduced correlation between the scores of the Picture-Geloph<9> and the GELOPH<15> in Study 2 (as compared to Study 1) may, in part, also be explained by the selection procedure employed to generate the Picture-Geloph<9> in Study 1: the criteria used to identify the conceptually most valid items may have led to selecting foremost items with a large linkage to self-reported fear. That is, preferring such items that (a) elicited scores with a lower “starting point” (i.e., non-fearful individuals, as defined by the GELOPH<15>, on average had negative scores in the selection of items), and (b) with a linear increase across the different groups of self-reported gelotophobia, may have increased both the variance of the Picture-Geloph<9> score as well as the covariance between the total scores of the Picture-Geloph<9> and the GELOPH<15> in this sample. Hence, because of the idiosyncrasies of the different samples, a lower estimate of convergent validity should have been expected when cross-validating the Picture-Geloph<9> with Sample 2. Still, there was a substantial correlation between the two measures of gelotophobia in Study 2, and adding Sample 3 yielded stronger results (despite the fact that the total score was based on six to eight items only). Taking into account that the GELOPH<15> and the Picture-Geloph<9> are different types of methods for the assessment of gelotophobia (i.e., a self-report vs. a semi-projective test), the coefficient found for the estimation of convergent validity in Sample 2 still can be seen as sufficiently high (i.e., due to a common-method effect, the correlation between two questionnaires can be expected to be higher than the correlation between a questionnaire and a different method of assessment, such as an objective or semi-projective test, even if all have the same validity).

The findings of our studies indicate, one more time, that the assumptions of Ruch et al. (2009) were substantial: gelotophobes tend to respond differently than the normal population when faced with situations in which they potentially could be laughed at, ridiculed or otherwise be evaluated as deficient or ridiculous. As a basic extension of their findings, the present study reveals that with an increasing level of the fear, this bias becomes more evident. At the same time, these results demonstrate that the Picture-Geloph<9> can be instrumental in the assessment of the varying levels of gelotophobia.

### Limitations

The present study demonstrated that the cartoons that are used by the Picture-Geloph<9> are suitable to evoke valid responses in gelotophobes. However, the sampling of the stimuli from which they were selected (i.e., the pilot version of the Picture-Geloph) may neglect important aspects of the fear of being laughed at. As the situations involving laughter are highly ambiguous, they do not provide explicit evidence that the protagonist is actually being addressed by the laughter. The feeling of being laughed at, therefore, is the result of a paranoid tendency to relate laughter to oneself (cf. Platt et al., 2012). It would be interesting to also capture gelotophobes’ disproportionately negative reactions in situations in which the normal population would also feel that they were being laughed at.

As a further limitation, the rating of responses was based on plausible but yet untested theoretical assumptions and therefore there were several disputable decisions made in the assignment of responses to the rating scale values by the two raters. For example, depending of the situation depicted by the item, responses reflecting *anger* were either assigned to the “negative response, but not explicitly fearing laughter” (+1) or the “slight enjoyment/engagement” (-1) rating scale value. In pictures where there was no evidence for the target person being addressed by the laughing persons, anger was interpreted as a possible sign of paranoid sensitivity to ridicule and hence rated as a possible indicator of gelotophobia, whereas angry responses to pictures in which the target person was obviously addressed with criticism or an insult were considered as a “healthy” reaction (as opposed to internalizing, i.e., thinking one deserves being criticized for one’s funny looks or awkward posture) and rated with the “slight enjoyment/engagement” (-1) scale value. Such discrepancies were not observed for more extreme answers.

The rating of responses reflecting embarrassment and shame needs reconsideration too. Being embarrassed or ashamed as a consequence of “justified” ridicule can be considered a usual response. However, responses reflecting shame and embarrassment were construed as possible indicators of gelotophobia in the reference-coding catalog. Shott (1979) points out that embarrassment is originated by deficiencies of the self-*presentation*, whereas shame occurs when others view one’s self—*per se*—as deficient. In line with this distinction, Tangney et al. (1996) suggest “shame is associated with more global and enduring negative attributions about oneself, whereas embarrassment is tied to more transient, situation-specific failures and pratfalls” (p. 1258). That would explain why gelotophobes are prone to shame: they misinterpret the criticism conveyed by ridicule (or rather anything they misperceive as ridicule) in line with their global belief that something essential is inherently wrong with them. But why and when are gelotophobes supposed to be embarrassed then? Shott (1979) reasons that, when the self is believed to be deficient, this also leads to the subjective impression of an inadequate self-*presentation*. As the crucial point, the situations depicted by the Picture-Geloph do not provide explicit evidence that the protagonist was transgressing relevant social norms prior to occurring laughter or criticism (or even is being laughed at), which may most likely be the reason why non-gelotophobes tend to respond neutrally or with enjoyment and the expression of positive attributes (or otherwise fear-atypically) to the stimuli. For the given reasons, responses expressing shame and embarrassment were construed as indicators of a biased conviction to be or to appear deficient, inadequate or ridiculous and were rated equally as negative (“negative response, but not explicitly fearing laughter,” i.e., +1) responses.

More research is needed on the optimal administration of the test. The setting in the present study might have primed some of the responses. The participants came to the data collection website pre-informed about the fear of being laughed at, and they also first filled in the GELOPH which might have led to different answers compared to randomly recruited participants who filled in the Picture Geloph as the first instrument. Systematic studies are needed to estimate the effects of the priming; e.g., whether level of face value is affected. Likewise, more research is needed examining when the validity of the test is maximal; such studies might vary the drawing style, but it is also of interest to add filler items to lower the face value of the test. Validity information needs to be accumulated to allow deciding whether the test can also be used in individual testing. Finally, in the present study two coders were employed (albeit only one was used further on). A study is needed to find out the optimal number of coders and the optimal level of training to be able to give profound advice on the optimal scoring circumstances.

### Recommendations for Future Studies

As a recommendation for future studies, it would be desirable to further extend the range of methods for the assessment of gelotophobia, for example, by means of an objective test. An objective assessment of gelotophobia could be based on the empirical findings of the studies conducted so far. For example, it has been shown that gelotophobes respond differently in a variety of modalities (such as behavioral, physiological, emotional) when encountering different stimuli (such as hearing laughter, being teased, or judging faces; Platt et al., 2013; Papousek et al., 2014; Ruch et al., 2014b, 2015). An objective test does not have face validity, which may complement the assessment of gelotophobia with an instrument in which scores are not easily influenced by response bias. Furthermore, the responses were only rated in a quantitative manner in the present study. A qualitative analysis might allow for a deeper understanding of gelotophobia and could be applied to test theoretical assumptions.

### Possible Applications

The interrater agreement indicates that, with some training, the Picture-Geloph<9> can be adopted by everyone. The proposed cut-off points for the mean total score of the ratings of responses for the 9-item standard version of the test are suitable to classify subjects into the categories non-fearful, slightly, markedly and extremely fearful. Hence, the Picture-Geloph<9> can be suggested for both the use in larger investigations and as supplementary information in individual testing. The Picture-Geloph<9> may be preferred to the GELOPH<15>, especially when it is desired not to impose the preconceived characteristics of gelotophobia on the test taker in the way that a questionnaire does. Furthermore, in individual testing, individuals’ responses to the Picture-Geloph<9> might be subsequently used as a starting point for a diagnostic interview. For example, in a therapeutic context a clinician may first administer the Picture-Geloph<9> and subsequently go through the items and explore what made the patient provide the respective answers. It can be seen as advisable combining the Picture-Geloph<9> with, subsequently, the GELOPH<15> (this order of use may help not to prime individuals when answering the questionnaire), in order to safeguard diagnostic decisions against a selective method bias (e.g., when individuals have an acquiescent tendency answering the GELOPH<15>). The Picture-Geloph<9> and the coding manual can be retrieved from the first author of this paper.

### Conclusion

In the present paper, an additional diagnostic tool for the assessment of gelotophobia was evaluated within a large sample of gelotophobes. The proposed 9-item standard scale allows for an economic and valid assessment of the fear of being laughed at. Furthermore, the phenomenon of the fear of being laughed at was demonstrated by means other than subjective self-reports in its full spectrum.

## Author Contributions

WR initiated the project and designed the concepts, TP collected data, RB, TP, and WR designed the coding scheme, RB and RĎ coded the answers. All authors contributed to the writing of the manuscript, read it critically, and gave consent to its publication.

## Conflict of Interest Statement

The authors declare that the research was conducted in the absence of any commercial or financial relationships that could be construed as a potential conflict of interest.

## Footnotes

1 This approach is different from semi-projective grid techniques, which typically require the test-taker to rate predefined statements concerning ambiguous stimuli (mostly pictures; cf. Ziegler et al., 2007).

2 Materials and data are available upon request from the first author.

3 There is no doubt that an item which is not meeting these criteria can still be found as a good indicator of the fear of being laughed at. However, the conceptual validity can be seen as highest among those stimuli, which also according to the true meanings of the categories of the coding scheme elicit fearful responses mostly in gelotophobes but not in fear-free individuals (and fear-atypical responses mostly in fear-free individuals but not in gelotophobes). Nevertheless, items that are to difficult or to easy might average out and be useful if included together.

4 In the parallel analysis the eigenvalues of the factors were compared to the means of eigenvalues originating from principal components analyses of 100 datasets with random data generated by permutations of the raw data set. The eigenvalue of the first, but not the second factor, met the retention criterion, as the eigenvalue of the second factor did not exceed the mean (*M* = 1.28) and consequently also did not exceed the upper 95th percentile (1.37) of the distribution of eigenvalues of second factors retrieved from the random data sets.

5 To illustrate: If a person’s test score is “1,” this would indicate a slight fear of being laughed at. However, taking into account the imperfect reliability of the scale, this person’s true score may as well indicate the absence of the fear of being laughed at (as defined by the suggested cut-off points), i.e., the true score might as well be “-1” (i.e., “1” minus the CI of “2”).

6 The eigenvalue of the second factor (and consequently also the eigenvalue of the third factor) did not exceed the mean (*M* = 1.31) and consequently also did not exceed the upper 95th percentile (1.42) of the distribution of eigenvalues of second factors retrieved from the random data sets (see Study 1 for the details of the procedure).
